# Clinical outcomes and prognostic factors for gastric cancer patients with bone metastasis

**DOI:** 10.1186/s12957-016-1091-2

**Published:** 2017-01-06

**Authors:** Jota Mikami, Yutaka Kimura, Yoichi Makari, Junya Fujita, Tomoya Kishimoto, Genta Sawada, Shin Nakahira, Ken Nakata, Masaki Tsujie, Hiroki Ohzato

**Affiliations:** 1 Department of Surgery, Sakai City Medical Center, 1-1-1 Ebarajicho, Nishi-ku, Sakai City, 593-8304 Osaka Japan; 2 Department of Surgery, Kindai University Faculty of Medicine, 377-2 Onohigashi, Sayama City, 589-8511 Osaka Japan

**Keywords:** Gastric cancer, Bone metastasis, Prognostic factor

## Abstract

**Background:**

Bone metastasis due to gastric cancer is rare, and the clinical features have not been fully evaluated. We investigated the clinical features, treatment outcomes, and prognostic factors in gastric cancer patients with bone metastasis.

**Methods:**

We retrospectively collected data on 34 consecutive patients who were diagnosed radiologically with bone metastasis due to gastric cancer. We estimated the overall survival after the diagnosis of bone metastasis using the Kaplan-Meier product-limit method and evaluated which clinicopathological factors were associated with prognostic factors for survival using univariate and multivariate Cox proportional hazards regression models.

**Results:**

The treatment for the primary tumor was surgery in 16 patients (47.1%) and chemotherapy in 18 patients (52.9%). The median serum alkaline phosphatase (ALP) and lactate dehydrogenase (LDH) levels at the time of bone metastasis were 375.5 and 249 IU/L, respectively. Ten patients (29.4%) were diagnosed with bone metastasis and gastric cancer at the same time. The 6-month survival rate after the diagnosis of bone metastasis was 63.8%, and the median survival time was 227.5 days. Multivariate analysis revealed that metachronous metastasis (*p* = 0.035) and extraosseous metastasis (*p* = 0.028) were significant risk factors for poor survival.

**Conclusions:**

The prognosis of gastric cancer with bone metastasis was poor, and metachronous metastasis and extraosseous metastasis were shown to be poor prognostic factors. Serum ALP, LDH, and tumor markers are not always high, so aggressive diagnosis using appropriate modalities such as bone scan, MRI, or PET-CT may be necessary in routine practice even in asymptomatic patients.

## Background

Although the incidence of gastric cancer has decreased in developed countries, it is the second most common cancer worldwide and two thirds of cases are found in developing countries [1]. The main sites of metastasis of gastric cancer are the liver and lungs, and the incidence of bone metastasis due to gastric cancer is only 0.9–2.1% [2], although there may be many gastric cancer patients who have not been diagnosed with metastasis clinically since the reported frequency of bone metastasis in gastric cancer was 13.4–15.9% in an autopsy series [3].

The median survival times of gastric cancer patients with bone metastasis are 3–4 months after the detection of bone metastasis [4, 5]. Since bone metastasis can cause intractable pain leading to poor quality of life, appropriate treatment strategies are essential for the affected patients [2]. Although the clinical characteristics and poor prognostic factors have been reported, they are not well-defined [4–7]. In this study, we retrospectively examined the clinicopathological features, treatment outcomes, and prognostic factors for survival in gastric cancer patients with bone metastasis.

## Methods

This study was approved by the institutional review board at Sakai City Medical Center. We retrospectively collected data on 34 consecutive patients who were radiologically diagnosed with bone metastasis due to gastric cancer between January 2010 and December 2015. All tumors were histologically diagnosed as adenocarcinoma with the stomach, which was recognized as primary tumor. Bone metastases have been treated after the clinical diagnosis by CT, PET-CT, bone scintigraphy, or MRI and after confirming that there were no other suspicious cancers by enhanced CT imaging from the chest to the pelvis. Clinicopathological data, such as age at the diagnosis of bone metastasis, gender, the Eastern Cooperative Oncology Group (ECOG) performance status scale, symptoms at the diagnosis of bone metastasis, tumor localization, differentiation, clinical or pathological stage (according to the 14th edition of the Japanese classification of gastric carcinoma to determine pathological stage [8]) at initial diagnosis, treatment for primary tumor (surgery or chemotherapy), treatment for bone metastasis (chemotherapy, radiotherapy, or best supportive care), and the spread of bone metastasis, were determined from patient records. The numerical values of serum alkaline phosphatase (ALP), serum lactate dehydrogenase (LDH), carcinoembryonic antigen (CEA), carbohydrate antigen (CA) 19-9, and CA125 were obtained from tests performed at the time of the diagnosis of bone metastasis. When the bone metastasis was observed at the same time as the diagnosis of gastric cancer, we defined it as a synchronous pattern of bone metastasis, while a metachronous pattern of bone metastasis was defined as bone metastasis detected at any time after the diagnosis of gastric cancer. For some patients, only a computed tomography (CT) scan was used in the diagnosis because bone metastasis was evident, while many patients were diagnosed using a combination of bone scintigraphy, positron emission tomography (PET)-CT, and magnetic resonance imaging (MRI).

We estimated the overall survival after the diagnosis of bone metastasis using the Kaplan-Meier product-limit method. We also evaluated which clinicopathological factors were associated with prognostic factors for survival using univariate and multivariate Cox proportional hazards regression models. Statistical significance was set at *p* < 0.05. All statistical analyses were performed using SPSS Statistics software, version 19 (IBM Corp., Armonk, NY, USA).

## Results

The median age of the 34 patients at the time the bone metastasis was diagnosed was 66 years (Table 1). There were 26 male patients and 8 females, and 19 patients (55.9%) had undifferentiated adenocarcinoma. The treatment for the primary tumor was surgery in 16 patients (47.1%), and 10 of them had radical resection. Out of 18 patients (52.9%) who used chemotherapy for initial treatment, 2 patients had surgery after chemotherapy with S1 and cisplatin. Eleven patients (32.4%) had bone pain at the time the bone metastasis was diagnosed. The median serum ALP and LDH levels at the time of bone metastasis were 375.5 and 249 IU/L, respectively. To diagnose the bone metastasis, CT scan was used for 30 patients (29.4%), and 15 patients of them underwent bone scintigraphy (9 patients), MRI (7 patients), and PET-CT (1 patient) after CT scan (Two patients underwent both bone scintigraphy and and MRI).

**Table 1:** Patient demographics and pathologic features

Factors		Patient (*n* = 34)
Age (years)	Median (range)	66 (39–78)
Gender	Male	26 (76.5%)
	Female	8 (23.5%)
ECOG performance status	0–1	16 (47.1%)
	2–4	18 (52.9%)
Bone pain	Present	11 (32.4%)
	Absent	23 (67.6%)
Location	Upper 1/3	5 (14.7%)
	Middle 1/3	17 (50.0%)
	Lower 1/3	5 (14.7%)
	Whole stomach	7 (20.6%)
Histologic type	Differentiated	15 (44.1%)
	Undifferentiated	19 (55.9%)
Stage^a^	I	1 (2.9%)
	II	3 (8.8%)
	III	8 (23.5%)
	IV	22 (64.7%)
Treatment for primary tumor	Surgery	16 (47.1%)
	Chemotherapy	18 (52.9%)
ALP (IU/L)	Median (range)	375.5 (157–2743)
LDH (IU/L)	Median (range)	249 (117–1481)
CEA	Median (range)	8.6 (1.0–3508)
CA19-9	Median (range)	53.7 (0.6–1814.0)
CA125	Median (range)	20.3 (7.9–1099)
Diagnostic modality	CT	30 (88.2%)
	Bone scan	10 (29.4%)
	MRI	9 (26.5%)
	PET-CT	1 (2.9%)
Pattern of bone metastasis	Synchronous	10 (29.4%)
	Metachronous	24 (70.6%)
Number of bone metastases	Single	12 (35.3%)
	Multiple	22 (64.7%)
Extraosseous metastasis	Present	26 (76.5%)
	Absent	8 (23.5%)
Treatment of bone metastasis	Chemotherapy	26 (76.5%)
	Radiotherapy	5 (14.7%)
	Best supportive care	4 (11.8%)

*Abbreviations*: *ALP* serum alkaline phosphatase, *LDH* lactate dehydrogenase, *CEA* serum carcinoembryonic antigen, *CA* carbohydrate antigen, *CT* computed tomography, *MRI* magnetic resonance imaging, *PET* positron emission tomography

^a^Stage was according to the 14th edition of the Japanese classification of gastric carcinoma

Ten patients (29.4%) were diagnosed with bone metastasis and gastric cancer at the same time, and 26 patients (76.5%) had at least one other organ affected besides their bones. Of the 24 patients who had metachronous metastasis, the median interval from the diagnosis of gastric cancer to the detection of bone metastasis was 398 days (range, 43–1799, data not shown).

The treatment of the bone metastasis consisted of chemotherapy in 26 patients (76%), radiotherapy in 5 patients (15%), and 4 patients only received the best supportive care (12%). The most common sites of bone metastases were the thoracic vertebrae (55.9%), pelvic bones (41.2%), lumbar vertebrae (38.2%), and ribs (29.4%) (Table 2). Many patients were treated with chemotherapy, such as S1-based regimens, as first-line chemotherapy, and taxane-based or irinotecan-based regimens as second and subsequent chemotherapies, according to the recommendation of the Japanese Gastric Cancer Treatment Guidelines for patients who have progressive or recurrent gastric cancer [9]. Some patients were treated with radiotherapy for a localized tumor or local symptom relief.

**Table 2:** Site of bone metastasis

Site of bone metastasis	Patient (*n* = 34)
Thoracic vertebrae	19 (55.9%)
Pelvic bones	14 (41.2%)
Lumbar vertebrae	13 (38.2%)
Ribs	10 (29.4%)
Cervical vertebrae	6 (17.6%)
Calvarium	4 (11.2%)
Scapula	3 (8.8%)
Lower extremity	3 (8.8%)
Upper extremity	2 (5.9%)
Clavicle	1 (2.9%)
Sternum	1 (2.9%)

**Fig. 1:**
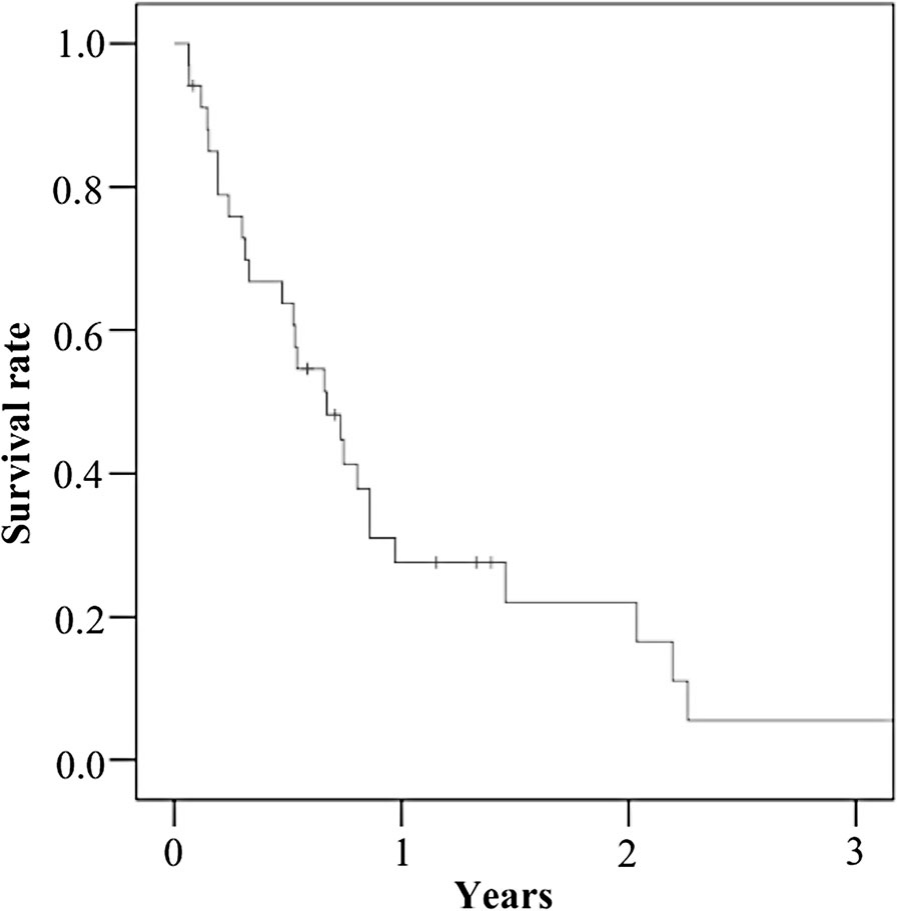
Overall survival of patients after the diagnosis of bone metastasis

The 6-month survival rate and the median survival time after the diagnosis of bone metastasis were 63.8% and 227.5 days, respectively (Fig. 1). A multivariate analysis revealed that metachronous metastasis (odds ratio 3.6; 95% confidence interval 1.1–11.7; *p* = 0.035) and extraosseous metastasis (odds ratio 4.1; 95% confidence interval 1.2–14.9; *p* = 0.028) were significant risk factors for poor survival (Table 3).

**Table 3:** Univariate and multivariate analyses of prognostic factors for survival

		Univariate analysis		Multivariate analysis	
		RR (95% CI)	*p* value	RR (95% CI)	*p* value
Age	≥75				
	<75	1.8 (0.25–13.7)	0.54		
Gender	Male				
	Female	1.1 (0.45–2.9)	0.78		
ECOG performance status	0–1				
	2–4	1.4 (0.57–3.4)	0.48	1.1 (0.41–2.8)	0.88
Bone pain	Absent				
	Present	2.0 (0.90–4.5)	0.091	2.7 (0.93–8.1)	0.068
Histologic type	Undifferentiated				
	Differentiated	1.1 (0.53–2.5)	0.73		
Stage^a^	I–III				
	IV	1.0 (0.46–2.2)	0.99		
Pattern of bone metastasis	Synchronous				
	Metachronous	2.8 (1.1–7.5)	0.038	3.6 (1.1–11.7)	0.035
Extraosseous metastasis	Absent				
	Present	1.1 (0.47–2.8)	0.77	4.1 (1.2–14.9)	0.028

*Abbreviations*: *CI* confidential interval, *ECOG* Eastern Cooperative Oncology

^a^Stage was according to the 14th edition of the Japanese classification of gastric carcinoma

## Discussion

In this study, the 6-month survival rate and the median survival time after the diagnosis of bone metastasis were 63.8% and 227.5 days, respectively. In univariate analysis, only pattern of bone metastases (synchronous vs metachronous) became an independent prognostic factor. The multivariate analysis was carried out using the pattern of bone metastases and variables pointed out to affect prognosis in past reports [2, 7]. As a result, metachronous metastasis and extraosseous metastasis were significant risk factors for poor survival.

In the period of this study, there were 622 patients who have been treated for gastric cancer for the first time in our hospital, and 34 of them have been diagnosed with bone metastasis. Most patients develop bone metastasis within 2 years of gastric surgery [3]. In this study, the median interval from the diagnosis of gastric cancer to the detection of bone metastasis was 398 days in the patients who had metachronous metastasis, and the median interval from the surgery to the detection of bone metastasis was 562 days. Bone metastases from gastric cancer were not unusual in a multicenter trial [10], and Turkoz et al. suggested that bone metastases should be considered during the follow-up of gastric cancer patients, even in the early period [3]. Patients may have relatively long-term survival if there is no extraosseous metastasis or local control for metastasis is possible, but it is difficult to diagnose bone metastasis because the majority of affected patients are asymptomatic and evaluations for bone metastases are not indicated in routine practice [7]. In our study, there were many cases that were discovered by chance during a routine CT examination. Only 32.4% of patients complained about symptoms, such as bone pain. In addition, serum ALP, LDH, or tumor markers were not always high, although there have been several reports that show such serum parameters were useful for diagnosing bone metastasis [11–13]. Ahn et al. suggested that an appropriate modality, such as bone scintigraphy, is required to assess bone metastasis at the time of the initial diagnosis and during follow-up observations [5].

Since there were no prospective studies of therapeutic regimens in gastric cancer patients with bone metastasis, the optimal chemotherapy regimens were unknown [2]. In this study, the treatment of metachronous bone metastasis differed depending on the judgment of the attending physician, and there had been various treatments before bone metastasis was recognized, while median survival time (MST) of patients who were treated metachronous bone metastasis with S1-based regimens or irinotecan-based regimens was significantly longer than MST of other patients (314 vs 87 days, *p* = 0.010). The Japanese Gastric Cancer Treatment Guidelines recommend S1-based chemotherapy for progressive or recurrent gastric cancer [9]. On the other hand, since bone metastasis can cause disseminated intravascular coagulation (DIC), the poor general condition of the patient or the presence of thrombocytopenia and severe anemia may make the patient ineligible for chemotherapy [14]. Hironaka et al. reported that sequential methotrexate and 5-fluorouracil chemotherapy resulted in a high rate of alleviation of DIC caused by bone metastasis from gastric cancer [15]. In addition, the pain management for patients with bone pain is important and radiation therapy may be quite effective [5]. In recent years, it has been reported that the incidence of epidermal growth factor receptor (EGFR) mutations in the bone metastases was high in the lung adenocarcinoma [16, 17]. Thus, EGFR tyrosine kinase inhibitor therapies could be effective for the type of adenocarcinoma. However, there are no reports about genetic mutations in the bone metastases due to gastric cancer. Identifying such mechanisms like gene mutations may lead to the development of future treatment.

In our study, the diagnosis of bone metastasis was left to the discretion of the attending physician and various modalities were used, so we could not evaluate which diagnostic methods were appropriate. In addition, the treatments varied depending on when the bone metastasis was detected. Therefore, further investigations are necessary.

## Conclusions

The prognosis of gastric cancer with bone metastasis was poor, and metachronous metastasis and extraosseous metastasis were shown to be poor prognostic factors. In addition, ALP, LDH, and tumor markers are not always high, so aggressive diagnosis using appropriate modalities such as bone scan, MRI, or PET-CT may be necessary in routine practice even in asymptomatic patients.

## Abbreviations

ALP: Alkaline phosphatase
CA: Carbohydrate antigen
CEA: Carcinoembryonic antigen
CT: Computed tomography
DIC: Disseminated intravascular coagulation
ECOG: Eastern Cooperative Oncology Group
LDH: Lactate dehydrogenase
MRI: Magnetic resonance imaging
PET: Positron emission tomography
